# Indigenous and faith healing in Ghana: A brief examination of the formalising process and collaborative efforts with the biomedical health system

**DOI:** 10.4102/phcfm.v11i1.2035

**Published:** 2019-07-22

**Authors:** Lily Kpobi, Leslie Swartz

**Affiliations:** 1 Department of Psychology, Stellenbosch University, Stellenbosch, South Africa

**Keywords:** mental health, faith healing, indigenous, collaboration, healers’ associations

## Abstract

**Background:**

Health seeking in many African countries typically involves making use of multiple healing systems, including indigenous and faith systems, as well as biomedical healthcare systems. These different systems have co-existed for many years in Africa, including in Ghana.

**Aim:**

In this article, we examine the formalising processes that non-biomedical healthcare in Ghana has undergone in postcolonial times. We first present a brief historical analysis of the process of organising indigenous medical systems into formal bodies. We then conclude by exploring collaborative efforts that have been undertaken between biomedical and non-biomedical health systems in Ghana.

**Method:**

A historical analysis of formalised indigenous healing systems in Ghana was done through an examination of relevant literature.

**Results:**

Formal groups of indigenous healers in Ghana who are organised into specific categories have undergone various transformations over the years. Evidence also exists of collaborative programmes developed with traditional healers in Ghana, although these have been largely for primary health partnerships. With regard to mental health collaborations, attempts at integration have been generally unsuccessful, with various factors identified as hindering successful partnerships.

**Conclusion:**

Indigenous healing is an important component of healthcare in Ghana. Collaboration between the different healthcare systems can be strengthened through accurate understandings of how key stakeholders are situated (and indeed situate themselves) in the conversation.

## Introduction

In a previous paper, the authors discussed the reported beliefs and practices of indigenous healers in Ghana, as well as the reported use (or preference) for indigenous healthcare by patients, with a specific focus on mental health. As we discussed previously, biomedicine in Ghana (together with Western notions of illness) was a component of colonialism.^1,2,3^ The colonial era also included a ban of indigenous healthcare practices and a suppression of indigenous cosmological beliefs.^1,3^ In this article, we present a brief history of the formalising process for traditional medical systems in Ghana. We also examine past and present collaborative efforts between biomedical systems and indigenous and faith healing systems in Ghana. Much of the literature on collaboration focuses on primary healthcare programmes, with few studies describing partnership with a specific mental health focus. Thus, in this article, while we make some reference to mental health, much of the available literature describes indigenous healing in general. As Hampshire and Owusu^4^ assert, most indigenous healers treat a range of illnesses based on specific cultural conceptualisations of ill health. As we discussed in our first paper, the systems of classification of some illnesses by indigenous healers is different from biomedical classifications. Similarly, conceptualisations of ill health vary.

There are different types of indigenous healers. Addy^5^ describes the categories of healers as consisting of those who use herbs, those who use spiritual psychicism and those who combine these methods. Therefore, healers may be classified as herbalists; diviners (called ‘fetish priests’ in Ghana), who use their knowledge of cultural norms and behaviours in their work;^6,7^ Muslim clerics, who use Islamic text and traditions to heal; and the Pentecostal Christian pastors, who use holy oils, holy water and other aids to perform healing. Within these categories, there may be subcategories (e.g. herbalists who specialise as traditional bonesetters). These healers typically work within communities where they are known to their patients.

### Ethical considerations

This article followed all ethical standards for a research without direct contact with human or animal subjects.

## Traditional medicine practice development in Ghana: From informal community practice to formalised care

As previously mentioned, indigenous healing processes were delegitimised and devalued during colonial times.^8^ In fact, under British rule in Ghana, the *Native Customs Regulation Ordinance* of 1878 banned indigenous healing practices outright.^9^ In addition to these official rules, the nature of cultural practice meant that indigenous systems evolved as cultures developed and thus were often context-specific. This made it difficult to create a comprehensive picture of indigenous healing practices in Ghana.

For a long time, there was limited knowledge on the work of indigenous healers in Ghana generally. The limited knowledge also made recognition and regulation of their work difficult to accomplish. With the attainment of political independence from colonial rule in the late 1950s, efforts were made to recognise and promote the work of indigenous healers in Ghana. In 1960, the first president of Ghana, Osagyefo Dr Kwame Nkrumah initiated the formal set-up of the Ghana Psychic and Traditional Healing Association, whose mandate was ‘to promote the study of herbalism and psychicism’ in Ghana for application in the public health sector.^10,11^ A further aim for the establishment of the association was to provide organisation to indigenous healers, as well as to lend some respectability to indigenous medicine practice in Ghana, in contrast to the disdain that had existed during colonisation. Thirdly, the association was expected to eventually facilitate indigenous healers working alongside ‘orthodox practitioners’ to treat illnesses, especially those for which there was no biomedical cure at the time.^9,11^

This inaugural group experienced many challenges in achieving its mandate as a result of differences in beliefs and orientation. Because of these challenges, several splinter groups emerged, such as the Plant Medicine Association, the Ghana Muslim Healers Association and the Ghana Psychic Healers Association. Individuals whose orientations were considered similar formed these splinter groups, and many of them still exist today. According to Mensah,^10^ presently there are six major indigenous healers’ association in Ghana, with numerous subgroups.

By 1999, the fragmented nature of organised traditional medicine practitioners’ groups had achieved very little by way of their intended mandate. Thus, in November 1999 further efforts were made to unite the different associations through the establishment of a new body called the Ghana Federation of Traditional Medicine Practitioners’ Associations (GHAFTRAM). The Ghana Federation of Traditional Medicine Practitioners’ Associations was formed to serve as a unifying body for the different categories of indigenous healers. They further served as a liaison between the healers and the Ministry of Health.

In 1991, the Ministry of Health established a Traditional Medicine Directorate (later renamed the Traditional and Alternative Medicine Directorate) as a formal division.^12^ A new law of the Food and Drugs Board (PNDC Law 305B) was also introduced in 1992 to regulate the manufacture and sale of herbal medications on the market. These, together with the establishment of GHAFTRAM and the development of the Ministry of Health’s *National Strategic Plan for Traditional Medicine Development* in 1999, facilitated the enactment of the *Traditional Medicine Practice Act (Act 575)* in 2000. This act mandated the formation of the Traditional Medical Practice Council (which was officially established in 2010) to license, regulate and oversee the work of traditional and alternative medicine practitioners in Ghana.^13^

The official recognition of alternative medicine in Ghana has therefore undergone some transformation over the years. Currently, the work of indigenous healers is governed by the Traditional and Alternative Medicine Division of the Ministry of Health, GHAFTRAM, the Food and Drugs Authority, and the Traditional Medical Practice Council.^5^ Specific to mental health, the newly passed *Mental Health Act (Act 846 of 2012)* refers to working with organised bodies (such as GHAFTRAM) to promote access to mental healthcare in Ghana.

Much of the effort at formal organisation of indigenous medicine has focused on the work of diviners and herbalists and, to a small extent, the Muslim healers. However, there is less official organisation for the work of Christian faith healers. Christian faith healers are predominantly Pentecostal or charismatic in orientation. As such, healing is considered an integral part of their religious expression. Although such churches are required to be registered with the Ghana Pentecostal and Charismatic Council, the healing centres (called prayer camps) are not registered separately as healthcare facilities, primarily because their activities may also include prayers for non-health problems. This presents difficulties for regulating and monitoring their work.

Although the efforts at organising alternative healers in Ghana have continued for some years, there are a significant number of healers who are not officially registered under any organisational body. Some reasons that have been suggested for this include the need for secrecy in the work of some categories of healers (e.g. the fetish priests) and, as discussed, the intertwining of core religious activities with health outcomes in some forms of practice.^14,15^

## Collaboration between biomedical institutions and indigenous healers in Ghana

There have been many calls for the integration of the different forms of healthcare in Ghana, as in other countries.^16^ As we have discussed, various factors account for the widespread use of alternative medicine in Ghana, including human resource constraints in the biomedical sector, as well as availability and perceived accessibility of indigenous and faith healers.^17^ As a result, there have been calls for and attempts at collaboration between the biomedical system and various alternative systems.^18^

One of the early recorded collaborations between biomedicine and herbal medicine in Ghana was in the Mampong-Akwapim District in the 1940s. This was an initiative of a medical doctor, Dr Oku Ampofo, who left government practice to set up a private practice with the aim of providing a recognised space for herbalists to practice alongside doctors.^19^ This partnership was considered quite successful, and through this collaboration Dr Ampofo compiled lists of medicinal plants and herbal remedies that were commonly used by herbalists in the region. This initiative is credited as being foundational for the establishment of the Centre for Scientific Research into Plant Medicine (CSRPM) in Ghana in 1975.^19^

Following the successful partnership in the Mampong-Akwapim area, the Ministry of Health, together with the CSRPM and other local stakeholders, developed the Primary Health Training for Indigenous Healers programme (PRHETIH)^11^ in Techiman in the early 1980s. This programme aimed to provide biomedical primary healthcare training for indigenous healers to improve their methods. It sought to widen the collaboration between biomedicine and other healers and therefore included herbalists, priests, traditional birth attendants and traditional surgeons. Although the programme was met with much enthusiasm, an evaluation performed 10 years later showed that the healers’ methods had not changed.^20^ Aries et al.^21^ speculated that the PRHETIH programme failed to achieve its intended purpose because of the different pathophysiological orientations that existed between the different classes of healers, a difference that had not been taken into account when the PRHETIH programme was developed. As Kpobi and Swartz^22^ explained, the beliefs about what constituted an illness differed based on the cultural and religious orientation of the healers. Without acknowledging this difference, integrating the two systems of care will be difficult. Konadu^6^ appeared to agree with this assertion, referring to the calls for integration as ‘an illusion’. According to him, the unequal political and cultural power relations would result in the forceful assimilation of one system into the other, leading to the eventual disappearance of the weaker group – most likely, the indigenous healers.^6,23^

Despite the perceived failure of the PRHETIH programme, other attempts at establishing collaborations have been reported.^24,25,26,27^ In 2011, the Ministry of Health, in a bid to begin integration of herbal and biomedical facilities, undertook a pilot study that introduced herbal units at hospitals. Small herbal medicine units were established in 17 hospitals across the country to provide patients with the option of purchasing certified herbal remedies.^28^ In reviewing this programme in one hospital in Kumasi 5 years later, Boateng et al.^28^ reported that the herbal and biomedical sectors at that hospital were running parallel to each other rather than being integrated with each other. Further, only a few patients were aware of the presence of the herbal unit. The authors speculated that this was likely because of the absence of clear policies and guidelines on referral between the two groups of healthcare providers.^28^ Thus, the different units existed separately, without working together as had been desired.

These reports are examples of collaborative programmes that were developed largely for primary health partnerships with traditional healers. With regard to mental health collaborations, attempts at integration have also been generally unsuccessful. Exploring the factors that hinder or promote partnerships between indigenous medicine practitioners and biomedical practitioners, Ae-Ngibise et al.^17^ observed that mutual distrust and scepticism, limited knowledge about practices and concerns about human rights abuses were cited by different stakeholders as important barriers that have prevented successful collaborations between mental health professionals and indigenous or faith healers. The stakeholders in this study included indigenous healers, biomedical practitioners and policymakers. Stakeholders in other studies^29^ expressed similar sentiments. For instance, Doku et al.^30^ reported comparable stakeholder views but also included inadequate policy implementation as a factor that affected integration of mental health services in Ghana. Awenva et al.^31^ identified barriers to policy implementation, including a lack of political interest in mental health, inadequate policy dissemination and an absence of research-based evidence for reform. The participants further advocated for collaborative efforts to be more intersectoral by including the educational, legal and development sectors of the country in order to be successful.

Osafo^32^ similarly examined the possibility of a collaborative network between mental health professionals and religious leaders in Accra. In addition to the reasons we have already cited, religious healers also decried biomedicine’s aversion to creating space for spiritual conditions, resulting in help-seeking stigma and territoriality. In order to achieve the desired integration of mental health service in Ghana, Osafo^32^ proposed a task-sharing model that incorporates a mutual appreciation for the work of the other.

This desire for mutual appreciation has been reported by indigenous and faith healers in other studies. There was a general perception among healers that biomedical professionals regarded them with some level of disdain, often dismissing their beliefs and methods.^17,33,34^ In one study, staff at prayer camps indicated a keen interest in working with the biomedical field to provide technical and infrastructural support (such as medication) but desired to do so only if they were treated with respect.^35^ In a related study, Ofori-Atta et al.^36^ worked with one prayer camp to provide biomedical care in addition to the standard spiritual care to a randomised sample of patients at the camp. According to the authors, the goal was to show staff at the camp the effectiveness of biomedical methods and to encourage collaboration between the two systems of care. Although the patients who were given medical treatment had better outcomes in the short term than their control counterparts, the authors found no significant change in the methods and attitudes of the staff at the camp. This lends support to the arguments of authors such as Read^37,38^ that indigenous and faith healers are aware of the usefulness of biomedical medications for short-term relief of symptoms but do not perceive biomedicine to provide better long-term outcomes.

In addition, the methods employed by Ofori-Atta and colleagues^36^ did not appear to seek an understanding of the prayer camp methods; instead, they appeared to simply demonstrate biomedicine’s prowess to the religious healers. While this was a unique initiative in the drive for collaboration, it echoes Konadu’s^6^ sentiments about the unequal power relations making integration difficult. In this study too, the prayer camp staff appeared to recognise the benefits of medication but felt that it did not provide the expected outcomes in the long run. Other studies with biomedical workers have shown similar attitudes towards the work of faith healers. The healthcare staff often recognised the usefulness of the spiritual engagement that religious healers employed but were concerned about what they perceived as subjective and unstandardised methods, some of which were considered abusive.^17,35^

Examining the role of indigenous and faith healers’ perceived power in the treatment of mental illness in Ghana, Read^38^ argued that a critical element to be considered in the dialogue on integrating mental health services was the practitioners’ beliefs surrounding the power of each system of care to heal the patient. Collaboration between the different systems would need to take into consideration the contested notions of healing power held by different practitioners, in order to be successful. Read^38^ reasoned that indigenous and faith healers often position themselves as possessing healing power that can provide longer-lasting solutions to patients’ problems than biomedicine. On the other hand, biomedicine was perceived by the healers to possess recognition and legitimacy in the national health framework.

Taking this assertion a step further, Kpobi and Swartz^39^ analysed the perceptions of power and positioning of different categories of indigenous healers, as well as how these self-perceptions influenced the willingness to collaborate with biomedicine. They identified that healers who perceived themselves as possessing abilities that were most powerful for providing healing (e.g. pastors and herbalists) were also most interested in collaborating with other systems of care. The authors argued that this was possibly because of these healers’ desire for recognition and formal acceptance, such as that biomedical systems appear to possess. On the other hand, those healers who considered themselves merely as conduits of a higher power did not desire formal collaboration. These healers were arguably not desirous of formal recognition because they believed their work was not a reflection of their own abilities but rather a reflection of the power of the deity they represented. Such competing ideas about power and place in the healing hierarchy would influence the willingness to collaborate. In order to achieve the desired collaboration, there needs to be recognition of the differing illness beliefs of the healers, as well as their different positioning with respect to healing power and their willingness to collaborate.

## Conclusion

The field of alternative medical care in Ghana has undergone various transformations over the years. Despite the many processes that have occurred, non-biomedical healthcare remains a popular option for a significant number of Ghanaians.^40^ Despite this popularity, many attempts to establish integrated healthcare that affords patients access to both biomedical and non-biomedical options have seen little success. Furthermore, the continued scepticism towards indigenous practices has resulted in difficulties in regulating, monitoring and improving indigenous care. Although initial collaboration programmes may have stalled, they may present valuable lessons on the way forward. In order to attain effective collaborative care in all spheres of healthcare, programmes and policies must be built on accurate understandings of how key stakeholders are situated (and indeed situate themselves) in the conversation.
